# Evaluation of the biodistribution and radiation dosimetry of the ^18^F-labelled amyloid imaging probe [^18^F]FACT in humans

**DOI:** 10.1186/2191-219X-3-32

**Published:** 2013-04-24

**Authors:** Miho Shidahara, Manabu Tashiro, Nobuyuki Okamura, Shozo Furumoto, Katsutoshi Furukawa, Shoichi Watanuki, Kotaro Hiraoka, Masayasu Miyake, Ren Iwata, Hajime Tamura, Hiroyuki Arai, Yukitsuka Kudo, Kazuhiko Yanai

**Affiliations:** 1 Division of Medical Physics, Tohoku University School of Medicine, 2-1 Seiryo-machi, Aoba-ku, Sendai, 980-8575, Japan; 2 Division of Cyclotron Nuclear Medicine, Cyclotron Radioisotope Center, Tohoku University, Sendai, 980-8578, Japan; 3 Department of Pharmacology, Tohoku University School of Medicine, Sendai, 980-8575, Japan; 4 Department of Geriatrics and Gerontology, Division of Brain Sciences, Institute of Development, Aging and Cancer, Tohoku University, Sendai, 980-8575, Japan; 5 Division of Radiopharmaceutical Chemistry, Cyclotron Radioisotope Center, Tohoku University, Sendai, 980-8578, Japan; 6 Clinical Research, Innovation and Education Center, Tohoku University Hospital, Sendai, 980-8574, Japan

**Keywords:** Positron emission tomography, Radiation dosimetry, Amyloid imaging, MIRD, [^18^F] FACT

## Abstract

**Background:**

The biodistribution and radiation dosimetry of the ^18^F-labelled amyloid imaging probe ([^18^F] FACT) was investigated in humans.

**Methods:**

Six healthy subjects (three males and three females) were enrolled in this study. An average of 160.8 MBq of [^18^F] FACT was intravenously administered, and then a series of whole-body PET scans were performed. Nineteen male and 20 female source organs, and the remainder of the body, were studied to estimate time-integrated activity coefficients. The mean absorbed dose in each target organ and the effective dose were estimated from the time-integrated activity coefficients in the source organs. Biodistribution data from [^18^F] FACT in mice were also used to estimate absorbed doses and the effective dose in human subjects; this was compared with doses of [^18^F] FACT estimated from human PET data.

**Results:**

The highest mean absorbed doses estimated using human PET data were observed in the gallbladder (333 ± 251 μGy/MBq), liver (77.5 ± 14.5 μGy/MBq), small intestine (33.6 ± 30.7 μGy/MBq), upper large intestine (29.8 ± 15.0 μGy/MBq) and lower large intestine (25.2 ± 12.6 μGy/MBq). The average effective dose estimated from human PET data was 18.6 ± 3.74 μSv/MBq. The highest mean absorbed dose value estimated from the mouse data was observed in the small intestine (38.5 μGy/MBq), liver (25.5 μGy/MBq) and urinary bladder wall (43.1 μGy/MBq). The effective dose estimated from the mouse data was 14.8 μSv/MBq for [^18^F] FACT.

**Conclusions:**

The estimated effective dose from the human PET data indicated that the [^18^F] FACT PET study was acceptable for clinical purposes.

## Background

### Amyloid beta imaging

Deposits of amyloid β (Aβ) plaque are one of the pathological observations in patients with Alzheimer's disease (AD); Aβ deposition progresses at an earlier point than the current clinical diagnostic point for this disease [1]. For earlier diagnosis of AD and the evaluation of treatment efficacy, *in vivo* amyloid imaging using positron emission tomography (PET), which provides quantitation and visualisation of Aβ deposition in the brain, is useful. Therefore, several Aβ-binding probes dedicated for PET imaging have been developed [2,3].

Most of these PET Aβ ligands are ^11^C-labelled compounds (physical half life (*T*_1/2_), 20 min), and ^18^F-labelled agents are being increasingly investigated owing to their long half life (*T*_1/2_, 109.7 min). The long *T*_1/2_ of ^18^F enables several PET scans to be carried out from a single synthesis of labelled agent and also enables its commercial distribution to any PET facility. On the other hand, the longer the *T*_1/2_ of the radioisotope gets, the greater is the radiation dose exposure for the PET subject for the same administered dose of radioligand.

### Importance of radiation dosimetry

For subjects undergoing PET, internal radiation exposure is inevitable, and the radiation dose delivered is proportional to the level of radioactivity of the injected radioligand and the number of injections. In the case of amyloid imaging, subjects often have multiple PET scans for diagnostic or therapeutic longitudinal monitoring of Aβ aggregation in the brain. Therefore, estimation of the radiation dose exposure from each PET radioligand and the use of well-balanced PET scan protocols taking into consideration subject risk and benefit are important.

Estimation of the internal radiation dose requires a time series measurement of the biodistribution of the injected radioligand. There are two ways to establish the biodistribution of a radioligand in humans: one is to extrapolate from data obtained in animal experiments [4] and the other is to use data from a clinical whole-body PET study [5]. Data extrapolated from animal experiments have been used to estimate clinical radiation dose. However, Sakata et al. reported that in some radioligands, there were considerable differences in organ dose or kinetics between human and animal experiments and that a whole-body PET study would be desirable for the initial clinical evaluation of new PET radioligands [6].

### Previous biodistribution and dosimetry study for PET amyloid imaging

Recently, radiation dose exposures from several PET amyloid imaging agents have been reported using clinical whole-body PET scans. One of the popular amyloid ligands, Pittsburgh compound B ([^11^C]PIB), has been extensively investigated with regard to its kinetics in the human body, and its effective radiation dose was found to be 4.74 μSv/MBq on average [7]. For ^18^F-labelled PET amyloid radioligands, effective doses in humans have been reported as follows: ^18^F-AV-45, 13 and 19.3 μSv/MBq [8,9]; ^18^F-GE067, 33.8 μSv/MBq [10]; and ^18^F-BAY94-9172, 14.7 μSv/MBq [11].

### Aim of the present study

Fluorinated amyloid imaging compound ([^18^F]FACT) is an ^18^F-labelled amyloid imaging agent developed at Tohoku University [12]. Kudo and colleagues at this university have previously developed a ^11^C amyloid imaging agent named [^11^C]BF-227 [3]. [^18^F]FACT is derived from [^11^C]BF-227 by reducing its lipophilicity in order to reduce the nonspecific binding in the brain; AD patients showed significantly higher uptake of [^18^F]FACT in the neocortex region relative to controls [12]. However, the biodistribution of [^18^F]FACT in humans has not yet been investigated.

In the present study, the radiation dosimetry and biodistribution of [^18^F]FACT was investigated in healthy elderly subjects who are the target group for PET amyloid imaging. In order to determine the discrepancy in the estimated radiation dose between human and animal experiments, biodistribution studies in mice involving [^18^F]FACT were also conducted.

## Methods

### Subjects

PET studies were performed in three healthy male and in three healthy female volunteers (mean age ± standard deviation (SD), 76.3 ± 3.2 years). Subject characteristics are shown in Table 1. Both height and weight varied over a wide range (146 to 175 cm and 39 to 74 kg, respectively). All subjects were Japanese and were free of somatic and neuropsychiatric illness, as determined by clinical history and physical examination; one male subject (no. 1) had undergone a previous surgical operation involving gallbladder removal.

**Table 1:** Information regarding the human subjects

	**Sex**	**Age (years)**	**Height (m)**	**Weight (kg)**	**BMI (kg/m**^**2**^**)**	**History**
Subject number						
1	M	77	1.59	61.2	24.2	Surgical removal of gallbladder
2	M	78	1.62	65	24.8	-
3	M	77	1.75	74	24.2	-
4	F	70	1.46	39	18.3	-
5	F	77	1.56	60.2	26.1	-
6	F	79	1.55	56	23.3	-
Mean ± 1 SD		76.3 ± 3.2	1.58 ± 0.75	59.2 ± 11.6	23.5 ± 2.7	

This study was approved by the Ethics Committee on Clinical Investigations of Tohoku University School of Medicine and was performed in accordance with the Declaration of Helsinki. Written informed consent was obtained from all subjects after a complete description of the study had been made.

### Radiochemistry and radioligand purity

**Figure 1:**
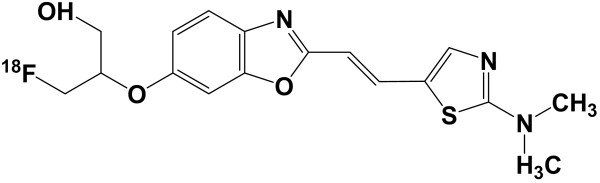
Chemical structure of ^18^F-FACT.

Figure 1 shows the chemical structure of ^18^F-FACT. The radiochemical purity of the radioligand in the present clinical study ranged from 97.8% to 98.7% (mean ± SD, 98.33 ± 0.42%). The specific radioactivity ranged from 30.6 to 347.7 GBq/μmol at the time of injection (mean ± SD, 139.9 ± 116.2 GBq/μmol).

### PET study

All whole-body PET studies were performed using a SET-2400W scanner (Shimadzu Inc., Kyoto, Japan) in two-dimensional (2D) mode [13]. The PET scanner acquired 63 image slices at a centre-to-centre interval of 3.125 mm and had a spatial resolution of 3.9 mm full width at half maximum (FWHM) and a *Z*-axis resolution of 6.5 mm FWHM at centre field of view [13].

**Figure 2:**
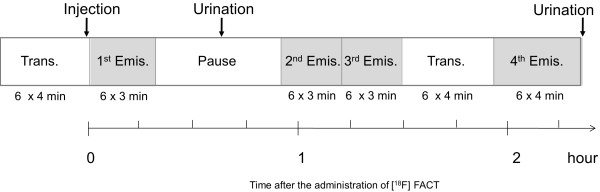
**Overview of PET scan protocols.** Four emission scans and two transmission scans (before and intermediate emission scans) with a ^68^Ge/^68^Ga source were performed. In particular, the second transmission scan was performed using a post-injection transmission scan.

An overview of scan protocols is shown in Figure 2. Four emission scans and two transmission scans (before administration and intermediate emission scans) using a ^68^Ge/^68^Ga source were performed, with the exception of subject no. 1 who had three emission scans. In the present series of PET studies and in other research projects, a 15-min PET brain scan using three-dimensional (3D) mode was performed after the first emission scan. At 2 min after intravenous administration of 142 to 180 MBq [^18^F]FACT (mean ± SD, 160.8 ± 14.8 MBq; injection mass, 0.77 ± 0.66 ng), a series of whole-body PET scans were performed. The schedule for the first and second transmission scans and the first, second, third and fourth emission scans was as follows: 6 positions × 4 min (24 min), 6 positions × 4 min (24 min), 6 positions × 3 min (18 min), 6 positions × 3 min (18 min), 6 positions × 3 min (18 min), and 6 positions × 4 min (24 min), respectively. The starting time of the second emission scan was different for each subject and was on average 55 min after the start of injection with a 5-min SD. The time gap between bed positions was 5 s. All emission data were reconstructed using OS-EM with iteration 16 and subset 2 after attenuation correction. Scatter correction was not performed because of the use of 2D mode data acquisition. The cross calibration factor of the scanner (Bq per ml/cps per voxel) was determined once per week using a cylindrical water phantom (25-cm length and 20-cm inner diameter) filled with ^18^F solutions and by measuring the sample activity of the ^18^F solutions at the well counter (BSS-3: Shimadzu Co., Ltd., Kyoto, Japan) [14].

Urination was controlled before, after and during the series of PET studies. In particular, during (15 min after the end of the first emission scan) and after the PET scans, subjects were asked to void. The volume and radioactivity levels of their urine samples were measured using a calibrated well counter.

### MRI study

All subjects underwent T1-weighted magnetic resonance imaging (MRI) scans using a Signa 1.5-T machine (General Electric Inc., Milwaukee, WI, USA) within a week of the PET scans. For each position (brain, chest, abdomen and epigastric region), individual T1-weighted scans with a voxel size of 1.875 × 1.875 × 6.000 mm (TR = 460 ms, TE = 14 ms, image matrix = 256 × 256 × 40) were obtained with subjects holding their breath.

### Dosimetry

The Medical Internal Radiation Dose committee of the Society of Nuclear Medicine developed the algorithm to calculate absorbed dose *D* (the energy deposited per unit mass of medium (Gy)) in organs. The basic idea is that radiation energy from the radioisotope in the source organ is absorbed in the target organs, and the algorithm requires the net accumulated radioactivity in source organs as an input [15]. A PET scan contributes to quantitative knowledge on the whole-body distribution of radioisotope. In the present study, the accumulated activity in source organs was derived from PET measurements and the organ volumes of the reference male or female. The mean absorbed dose to the *k*th target organ is defined as follows:

(1) $$\bar{D}\left(r_{k}\right)=\sum_{h}{\tilde{A}}_{h}\;\times \;S\left(r_{k}\leftarrow r_{h}\right)=\sum_{h}A_{0}\;\times \;\tau_{h}\;\times \;S\left(r_{k}\leftarrow r_{h}\right)\text{,}\;\tau_{h}=\frac{{\tilde{A}}_{h}}{A_{0}}$$

where *S*(*r*_*k*_ ← *r*_*h*_) is the absorbed dose in the *k*th target organ per unit of accumulated activity in the *h*th source organ, called the *S* value. ${\tilde{A}}_{h}$ is the number of disintegrations in the *h*th source organ, *A*_0_ is the injected dose, and *τ*_*h*_ is the time-integrated activity coefficient in the *h*th source organ (equal to the number of disintegrations per unit activity administered). The effective dose *E* (Sv), as defined by the International Commission on Radiological Protection (ICRP) 60 [10], was obtained using the following equation:

(2) $$E=\sum_{i}Q\;\times \;w_{i}\;\times \;D_{i}$$

where *D*_*i*_ is the absorbed dose of the *i*th target organ, *w*_*i*_ is the weighting risk factor in the *i*th target organ, and *Q* is the quality factor (*Q* = 1 for β- and γ-rays).

### Regions of interest

The number of source organs used for region-of-interest (ROI) drawing was 19 for male and 20 for female subjects. A detailed list of source organs is shown in Table 2. Two nuclear medicine physicians manually drew the ROIs using PMOD version 3.1 (PMOD Technologies, Zurich, Switzerland). All individual PET images and MRI images were co-registered to the first individual PET images using a rigid matching module of the same PMOD with a dissimilarity function of normalised mutual information (for MRI-to-PET cases) and the sum of the absolute difference (for PET-to-PET cases) algorithms.

**Table 2:** [^18^F]FACT time-integrated activity coefficients in the source organs

**Organ**	**Human (MBq-h/MBq)**							**Mouse (MBq-h/MBq)**
	**Mean ± 1 SD**	**Subject 1**	**Subject 2**	**Subject 3**	**Subject 4**	**Subject 5**	**Subject 6**	
Adrenal gland	5.38E−04 ± 2.98E−04	9.40E−04	8.40E−04	5.20E−04	3.70E−04	4.00E−04	1.60E−04	-
Brain	4.20E−02 ± 8.44E−03	5.41E−02	3.68E−02	3.53E−02	4.87E−02	4.42E−02	3.26E−02	6.99E−03
Breast	8.40E−03 ± 4.37E−03	1.14E−03	8.25E−03	5.59E−03	1.16E−02	1.19E−02	1.19E−02	-
Gallbladder content^a^	2.22E−01 ± 1.05E−01	-	1.49E−01	2.27E−01	3.88E−01	1.16E−01	2.31E−01	-
Lower large intestine content	2.12E−02 ± 2.03E−02	5.91E−02	1.06E−02	4.80E−03	2.27E−02	2.40E−02	5.96E−03	-
Small intestine content	8.78E−02 ± 1.08E−01	7.40E−02	3.74E−02	3.34E−02	3.06E−01	3.36E−02	4.25E−02	1.22E−01
Stomach content	6.71E−03 ± 2.28E−03	5.22E−03	6.23E−03	9.29E−03	9.84E−03	4.78E−03	4.87E−03	-
Upper large intestine content	2.55E−02 ± 1.89E−02	1.48E−02	4.88E−02	1.01E−02	4.99E−02	2.04E−02	8.85E−03	-
Heart content	1.12.E−02 ± 1.51E−03	1.24E−02	1.13E−02	1.02E−02	1.30E−02	1.15E−02	8.83E−03	3.95E−03
Heart wall	7.50E−03 ± 1.84E−03	4.63E−03	1.00E−02	8.84E−03	6.75E−03	7.28E−03	7.49E−03	2.39E−03
Kidney	1.34E−02 ± 3.27E−03	1.32E−02	1.16E−02	1.53E−02	1.89E−02	9.59E−03	1.20E−02	9.34E−03
Liver	4.92E−01 ± 1.05E−01	6.28E−01	5.85E−01	4.34E−01	5.15E−01	3.42E−01	4.49E−01	1.69E−01
Lung	3.55E−02 ± 1.16E−02	3.78E−02	5.31E−02	4.36E−02	2.46E−02	2.33E−02	3.08E−02	1.17E−02
Muscle	4.66E−01 ± 3.73E−01	9.43E−01	5.47E−01	7.97E−01	5.40E−02	4.12E−01	4.34E−02	1.57E−01
Ovary^b^	5.53E−04 ± 3.79E−05	-	-	-	5.70E−04	5.10E−04	5.80E−04	-
Pancreas	4.13E−03 ± 9.75E−04	6.10E−03	3.62E−03	3.93E−03	3.81E−03	3.55E−03	3.76E−03	-
Red marrow	3.98E−02 ± 4.33E−03	4.02E−02	3.83E−02	3.79E−02	4.59E−02	4.29E−02	3.34E−02	1.61E−02
Spleen	5.41E−03 ± 1.74E−03	8.64E−03	4.88E−03	3.83E−03	6.01E−03	4.77E−03	4.30E−03	1.28E−03
Testis^c^	5.77E−04 ± 4.67E−04	7.00E−04	6.10E−05	9.70E−04	-	-	-	-
Thyroid	3.53E−04 ± 1.55E−04	6.30E−04	3.70E−04	3.60E−04	2.40E−04	1.80E−04	3.40E−04	-
Urinary bladder contents	2.26E−02 ± 8.36E−03	1.70E−02	2.69E−02	1.55E−02	3.63E−02	2.49E−02	1.51E−02	6.56E−02
Uterus/uterine wall^b^	4.46E−03 ± 1.90E−03	-	-	-	6.42E−03	2.63E−03	4.33E−03	-
Remainder of the body	1.17 ± 3.63E−01	7.24E−01	1.06	9.48E−01	1.08	1.51	1.69	2.08

Averaged time-integrated activity coefficient (MBq-h/MBq) for the source organs (*n* = 6) from the whole-body PET data (*n* = 6) from experiments involving human subjects of [^18^F]FACT and mice of [^18^F]FACT. ^a^Averaged value among five subjects excluding subject no. 1. ^b^Average time-integrated activity coefficient among female subjects (*n* = 3). ^c^Average time-integrated activity coefficient among male subjects (*n* = 3).

For visceral organs with extremely high uptake (liver and gallbladder), individual ROIs were defined at a fixed emission scan with about a 40% threshold against the maximum counts (first emission for the liver and third or fourth emission for the gallbladder). Then the ROI was applied to the other emission images with minor adjustment of its location or shape. For the intestines, if specific high uptake was observed, individual ROIs were defined on each time frame of the PET images with about a 10% threshold. If there was no specific high uptake in the intestines, and uptake could be regarded as uniform, individual ROIs were drawn around the corresponding area.

In order to obtain a typical radioactivity concentration within organs with less location mismatch between PET and the co-registered MRI images (brain, breast, heart wall, heart contents, kidney, liver, lung, muscle, bones, spleen and thyroid), individual ROIs were drawn on co-registered MRI images. For other low-uptake organs (adrenal gland, stomach contents, pancreas, ovary, uterus and testis), individual ROIs were drawn on each time frame of the PET images and referred to the co-registered MRI images. To avoid a partial volume effect, the size of the ROI for these MRI available organs was made slightly smaller than the entire source organ. It should also be noted that all activities in vertebrae ROIs was assumed to be in the red marrow in the present study.

### Data analysis

Averaged time-activity curves for each organ were obtained using the ROI values from each subject's PET images. Because the PET images were decay-corrected at the start of each scan during the reconstruction procedure, the non-decay-corrected time-activity curves (*C*(t), Bq/ml) were re-calculated. During each whole-body emission scan, the bed position was moved from the foot to the head (six bed positions in total). However, we assumed that PET counts at all bed positions were acquired at the mid-scan time. Then, individual radioactivity concentration per injected dose *A*_0_ (Bq) was extrapolated into the percent injected dose (%ID) of the reference subject as follows:

(3) $$\%\mathrm{ID}{\left(t\right)}_{\mathrm{reference}}={\left(\frac{C\left(t\right)}{A_{0}}\right)}_{\mathrm{individual}}\times V_{\mathrm{reference}}$$

where *V* (ml) is the organ volume, and *V*_reference_ is *V* of the reference subject (we used a 70-kg adult male and 58-kg adult female as the male and female reference subjects) [16,17]. Even though some organs such as the intestine may change their volume over time, we used the reference subjects' organ volumes over the time period of the calculation of the %ID.

The time-integrated activity coefficient *τ* (Bq-h/Bq) in Equation 1 was obtained by fitting (%ID(*t*)) using a mono-exponential function and integrating from time zero to infinity. If the time-activity curve did not converge at the last PET scan (e.g. intestines and gallbladder), time-activity curves were fitted using two exponential functions, and then the area under the curve after the acquisition of the last image was calculated by assuming only physical decay of ^18^F and no additional biologic clearance to be conservative [10]. The time-integrated activity coefficient for urinary bladder content was calculated by applying the dynamic urinary bladder model [10] to the urine samples with a bladder voiding interval of 2 h. The decay-corrected cumulative activity for urine was fitted using the equation *A* × (1 − exp(−ln(2) × *t* / *τ*)), where *τ* is the biological decay and *A* is the fraction of activity released from the body. The sum of the time-integrated activity coefficient for the specific organs was subtracted from the time-integrated activity coefficient for the total body, which was calculated from the time integral of the decaying injected radioactivity. Then the residual of the subtraction was regarded as the time-integrated activity coefficient in the remainder of the body. All fitting procedures were undertaken using a mean fit of *R*^2^ of 0.93 ± 0.13.

Finally, the time-integrated activity coefficient *τ* (Bq-h/Bq) was used to calculate the absorbed dose, *D*, in Equation 1 and the effective dose, *E*, in Equation 2. Both kinetics calculations (fitting and integration) and dose estimation were performed using OLINDA/EXM software version 1.0 (Department of Radiology and Radiological Sciences Vanderbilt University, Nashville, TN, USA) [17].

### Animal experiments

The experimental protocols were reviewed by the Committee on the Ethics of Animal Experiments at Tohoku University School of Medicine and performed in accordance with the Guidelines for Animal Experiments issued by the Tohoku University School of Medicine. Estimated radiation dose of [^18^F]FACT in the human subjects calculated from mouse data sets was compared with those of [^18^F]FACT from human whole-body PET scans. An average dose of 1.4 MBq of [^18^F]FACT was intravenously injected into ICR mice (age, 6 weeks; average body weight, 30 g) without anaesthesia. In the [^18^F]FACT study, the mice were killed by cervical luxation at 2, 10, 30, 60 and 120 min ([^18^F]FACT) after administration (*n* = 4 at each time point). The masses of the blood, heart, lung, liver, spleen, small intestine, kidney, brain and urine samples were measured, and activity was also measured using a well counter. Thigh bone and muscle were also sampled. The average uptake of the radioligand into the male reference subject (70 kg) was extrapolated as follows [18]:

(4) $${\%\mathrm{ID}(t)}_{\mathrm{human}}={\left(\frac{\%\mathrm{ID}\left(t\right)}{{\mathrm{mass}}_{\mathrm{organ}}}\right)}_{\mathrm{mouse}}\times {\left({\mathrm{mass}}_{\mathrm{body}}\right)}_{\mathrm{mouse}}\times {\left(\frac{{\mathrm{mass}}_{\mathrm{organ}}}{{\mathrm{mass}}_{\mathrm{body}}}\right)}_{\mathrm{human}}$$

where the bodyweight of the mouse was assumed to be 30 for [^18^F]FACT.

Finally, in the same manner as in the human PET data analysis, time-integrated activity coefficients, absorbed doses and effective doses were calculated using the OLINDA/EXM software version 1.0. Sampled blood, thighbone and urine were regarded as heart contents, red bone marrow and urinary bladder contents, respectively.

## Results and discussion

### Biodistribution of [^18^F]FACT

**Figure 3:**
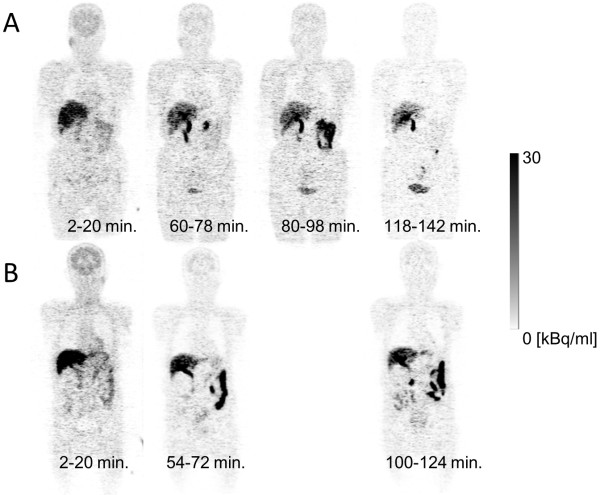
**Decay-corrected coronal radioactivity distributions.** Subject no. 5 (**A**) and subject no. 1 (**B**) at each PET measurement.

Figure 3A is the coronal PET image for a single female subject (no. 5) and demonstrates the typical biodistribution of [^18^F]FACT in the human body. The highest accumulations of this radioligand were observed in the gallbladder, liver, intestine and urinary bladder. For subject no. 1, [^18^F]FACT contained in the bile was excreted from the liver to the duodenum through the biliary tract (Figure 3B). The biodistribution pattern of [^18^F]FACT in human subjects showed a predominant hepatobiliary excretion, which is similar to what has been observed for other amyloid ligands, such as [^11^C]PIB, [^18^F]AV-45, [^18^F]GE067 and [^18^F]BAY94-9172 [7,8,10,11].

**Figure 4:**
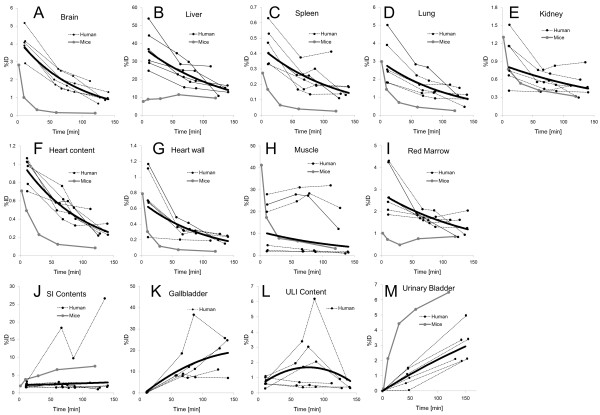
**Decay-corrected time-activity curves of %ID.** (**A**) Brain, (**B**) liver, (**C**) spleen, (**D**) lung, (**E**) kidney, (**F**) heart content, (**G**) heart wall, (**H**) muscle, (**I**) red marrow, (**J**) SI contents, (**K**) gallbladder, (**L**) ULI content and (**M**) urinary bladder for individual human (dashed black line) and averaged mice extrapolation (*n* = 4 at each time point, grey line). The solid black line indicates fitted curve using exponential function from all subjects' data points. The urinary bladder curve indicates accumulated activity excreted at all voiding/sampling moments.

Figure 4 shows the decay-corrected time-activity curve of the %ID for typical source organs (brain, liver, spleen, lung, kidney, heart content, heart wall, muscle, red marrow, small intestine contents, gallbladder, upper large intestine contents and urinary bladder) from the six volunteers and the mice experiments. A significant difference between the %ID from humans and mice was observed in the brain, liver, spleen, heart contents, red marrow and urinary bladder, and these differences propagated the different results regarding dose estimation. In human subjects, ^18^F uptake in the gallbladder contents and the intestines (Figure 4J,K,L) indicated larger individual variations in radioactivity uptake relative to other organs (e.g. the kidney as shown in Figure 4E). Radioactivity uptake in the upper large intestine showed propagation of both ligand kinetics and inter-subject variation from the gallbladder (Figure 4K,L). Scheinin et al. previously reported that inter-subject variation in ligand uptake ([^11^C]PIB) in the gallbladder may be due to the quality and quantity of post-injection food intake [7]. In the present study, the subjects drank water during the interval between the first and second PET scans. This may have been responsible for the increase in inter-subject variation regarding the gallbladder. Furthermore, because the gallbladder uptake in some subjects had declined or remained at a low level at the final time points, we assumed that there was only physical radioactive decay after the last PET scans. However, this assumption may have led to a conservative estimation of the absorbed dose.

**Figure 5:**
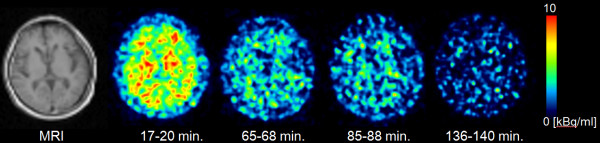
Decay-corrected brain PET images of subject no. 3 at different time points.

Figure 5 presents typical brain PET images obtained using [^18^F]FACT at different time points with an acquisition time of 3 min (first, second and third emission) and 4 min (fourth emission). There was no significant retention of [^18^F]FACT in the brain, and this may have been because the subject was normal.

### Estimated dose of [^18^F]FACT

The [^18^F]FACT time-integrated activity coefficients in the source organs are shown in Table 2, and the absorbed doses are shown in Table 3. The averaged time-integrated activity coefficient for the gallbladder, as shown in Table 2, was calculated among five subjects excluding subject no. 1; however, in the case of the averaged absorbed and effective doses, subject no. 1 was included (Table 3).

**Table 3:** Absorbed doses in the source organs

	**Human**			**Mouse**
	**All subjects (*n *= 6)**	**Male**	**Female**	
		**(*n *= 3)**	**(*n *= 3)**	
Organ				
Adrenal gland	1.96E01 ± 2.00	2.03E01	1.90E01	1.35E01
Brain	9.91 ± 1.82	8.95	1.09E01	4.17
Breasts	8.69 ± 2.55	6.68	1.07E01	9.90
Gallbladder wall	3.33E02 ± 2.51E02	2.16E02	4.50E02	1.68E01
Lower large intestine wall	2.52E01 ± 1.26E01	2.41E01	2.63E01	1.57E01
Small intestine	3.36E01 ± 3.07E01	2.07E01	4.64.E01	3.85E01
Stomach wall	1.61E01 ± 3.44	1.35E01	1.87E01	1.39E01
Upper large intestine wall	2.98E01 ± 1.50E01	2.36E01	3.59E01	1.83E01
Heart wall	1.62E01 ± 1.70	1.50E01	1.74E01	8.34
Kidneys	2.01E01 ± 4.30	1.85E01	2.17E01	1.32E01
Liver	7.75E01 ± 1.45E01	7.38E01	8.11E01	2.55E01
Lungs	1.46E01 ± 1.10	1.49E01	1.43E01	7.96
Muscle	1.03E01 ± 1.27	1.07E01	9.90	7.89
Ovary	1.67E01 ± 6.65	1.18E01	2.16E01	1.67E01
Pancreas	2.32E01 ± 3.11	2.17E01	2.47E01	1.45E01
Red marrow	1.31E01 ± 1.70	1.16E01	1.46E01	1.23E01
Osteogenic cells	1.60E01 ± 3.65	1.25E01	1.95E01	1.82E01
Skin	7.30 ± 1.39	5.99	8.60	8.70
Spleen	1.37E01 ± 2.48	1.27E01	1.48E01	7.83
Testis	7.32 ± 2.16	7.32	-	1.15E01
Thymus	1.00E01 ± 1.85	8.37	1.16E01	1.08E01
Thyroid	8.36 ± 1.38	8.86	7.86	1.10E01
Urinary bladder wall	2.23E01 ± 7.33	1.81E01	2.66E01	4.31E01
Uterus	1.67E01 ± 8.13	1.14E01	2.19E01	1.77E01
Total body	1.38E01 ± 1.63	1.22E01	1.53E01	1.22E01
Effective dose (μSv/MBq)	1.86E01 ± 3.74	1.64E01	2.09E01	1.48E01

Averaged absorbed dose estimates (μGy/MBq) for the target organs from the whole-body PET data (*n* = 6) from experiments involving human subjects of [^18^F]FACT and mice of [^18^F]FACT. Average absorbed dose for male subjects (*n* = 3).

High absorbed dose in humans was observed in the gallbladder (333 ± 251 μGy/MBq), liver (77.5 ± 14.5 μGy/MBq), small intestine (33.6 ± 30.7 μGy/MBq), upper large intestine (29.8 ± 15.0 μGy/MBq) and lower large intestine (25.2 ± 12.6 μGy/MBq). In mice, high absorbed doses were observed in the small intestine (38.5 μGy/MBq), liver (25.5 μGy/MBq) and urinary bladder wall (43.1 μGy/MBq) for [^18^F]FACT (Table 3).

The effective dose estimated from the human PET study was 18.6 ± 3.74 μSv/MBq. The effective doses of [^18^F]FACT estimated from the clinical PET studies among other ^18^F-labelled PET amyloid radioligands were as follows: [^18^F]AV-45, 13 and 19.3 μSv/MBq [8,9]; [^18^F]GE067, 33.8 μSv/MBq [10]; and [^18^F]BAY94-9172, 14.67 μSv/MBq [11]. For PET analysis of [^11^C]PIB, Scheinin et al. normalised the %ID using the ratio of individual and reference subjects' body weights (Equation 4) [7]. However, in the present study, we did not normalise the %ID data because there was a small difference between the effective dose with normalisation (17.6 ± 2.12 μSv/MBq) and the present effective dose (18.6 ± 3.74 μSv/MBq). Therefore, we concluded that body weight normalisation does not influence the effective dose.

The effective dose of [^18^F]FACT from the mouse experiments (14.8 μSv/MBq) was underestimated as compared with that from the human subject PET studies (18.6 μSv/MBq) (Table 3). This discrepancy corresponded to 0.76 mSv (2.96 and 3.72 mSv from mice and humans, respectively) while assuming an injected activity of 200 MBq as a clinically relevant dose. The underestimation of absorbed dose in the mouse gallbladder (20 times lower) and liver (3 times lower) relative to the human PET studies may have been responsible for the underestimation of the effective dose. High absorbed doses in the liver, gallbladder and small intestine of mice indicated that the biodistribution pattern of [^18^F]FACT in mice includes hepatobiliary excretion, as was observed in the PET scans involving human subjects. However, the estimated absorbed dose in the gallbladder was 20 times lower than the estimate from human subject data sets because we could not remove the gallbladder of the mouse. Therefore, to evaluate the effective dose of [^18^F]FACT in target organs, a whole-body PET scan of human subjects may be preferable as compared with the extrapolation from mouse experiments.

### Clinical applicability of [^18^F]FACT

The present whole-body PET study was performed using healthy elderly subjects and not patients with AD. Previously, Koole et al. speculated that if brain uptake of ^18^F amyloid ligand increased by a factor of three, this will only influence estimation of the effective dose within 1%; however, when the subject had taken medication that changed the function of the hepatic metabolism, the estimated effective dose will vary with a larger range [10].

In the present series of PET studies, brain PET scans using the 3D mode were performed between the first and the second emission scan. Therefore, the injected dose for 2D whole-body scans was set to lower level than usual, and the averaged injected activity of 160.8 MBq corresponded to a radiation dose of 2.99 mSv per single administration. With regard to the optimal injected activity that can ensure sufficient image quality for clinical use, the peak noise-equivalent counts ratio (NECR) is often used in its determination. It has also been reported that the peak NECR in 2D mode was not reached with an acceptable range of injected activity, whereas in 3D mode, there was a distinct maximum for the NECR for which the corresponding injected activity was based on patient height and weight [19]. For the scanner used in our study, the NECR peak in 3D mode was reached at 4.44 kBq/ml using an 8,000-ml phantom [13]. When the subject's height and weight were assumed to be 170 cm and 60 kg, respectively, this assumption corresponded to the optimal injected dose of about 260 MBq. In a real situation, there exists the effect of the activity outside the axial FOV, and the optimal injected dose would be much lower. Injected activity indicates radiation dose; for example, 200 MBq indicates a radiation dose of 3.72 mSv. ICRP 62 [20] recommended that the maximum radiation dose that causes a ‘minor to intermediate’ increase of risk levels while preserving social benefit levels that are ‘intermediate to moderate’ has an effective limit of 10 mSv/year [20,21]. Thus, the maximum injectable activity is 537.6 MBq [^18^F]FACT/year, and this injection dose limit allows two or three PET scans to be performed. Furthermore, amyloid imaging is mainly undertaken in elderly patients aged >50 years, even though for early detection of AD, patients aged <50 years will also have an amyloid PET scan. According to the guidance on medical exposures in medical and biomedical research by the European Commission [22], dose restrictions for patients aged over >50 years are not as strict as for younger patients. Therefore, considerably more multiple PET scans may be possible.

## Conclusions

The effective dose of the ^18^F-labelled amyloid imaging agent, [^18^F]FACT, was found to be acceptable for clinical study.

## Competing interests

The authors declare that they have no competing interests.

## Authors’ contributions

MS carried out the data analysis and interpretation and drafted the manuscript. TM, HT, YK and KY performed the study design and contributed to the intellectual discussion. NO, SF and RI performed the animal experiments and synthesis of PET probes. KF, SW, KH, MM and HA provided the clinical data. All authors read and approved the final manuscript.
